# Introducing Experimental Design Concepts Improves Biochemistry Undergraduates' Experimental Laboratory Competence and Confidence

**DOI:** 10.1002/bmb.70046

**Published:** 2026-03-14

**Authors:** Nathan D. Tivendale, Alyssa Van Dreumel, Peter G. Arthur

**Affiliations:** ^1^ School of Molecular Sciences, the University of Western Australia Crawley WA Australia; ^2^ Tasmanian Institute of Agriculture, University of Tasmania Launceston Tasmania Australia

**Keywords:** biochemistry, competence, confidence, critical thinking, experimental design, laboratory education, undergraduate education

## Abstract

Laboratory science education can provide a training ground for developing experimental design capabilities, the ability to critically assess a range of laboratory methods, and confidence in selecting, adapting, and performing laboratory techniques. However, traditional, fail‐safe, protocol‐based laboratories provide little opportunity for students to develop these cognitive abilities. We introduced experimental design elements into a second‐year undergraduate biochemistry laboratory. Using a guided inquiry model, we show that students improve their self‐perceived and externally measured ability to critically assess laboratory methods, design experiments, and adapt protocols (experimental laboratory competence) and gain confidence in adapting protocols and applying technical laboratory knowledge to new situations (experimental laboratory confidence). Furthermore, students on the whole reported enjoying engaging with experimental design.

## 1 Introduction

Thriving market economies depend upon training and education, so one of the aims of educational institutions is to produce competent and desirable graduates [1]. For science students, this education consists of theoretical knowledge acquisition and, in general, practical laboratory education [2]. The latter is an important training ground not only for gaining hands‐on experience of technical laboratory methods but also for developing experimental design capabilities, laboratory‐related critical thinking, and confidence in selecting, adapting, and performing laboratory techniques [3]. Laboratory classes present an opportunity for students to develop critical thinking skills such as analysis and evaluation.

Developing technical laboratory skills and the associated cognitive abilities are important components of science education [4, 5], because they are desirable to many employers (summarized in [4, 6]). However, traditional protocol‐based laboratory classes usually focus on the former (i.e., developing technical laboratory skills) and do not maximize the development of the latter (i.e., developing the associated cognitive abilities). Traditional laboratory classes, where students follow a set protocol, do not enable students to develop key employability skills, such as the ability to confidently design their own experiments [7] or recall laboratory techniques and adapt them to new challenges [8, 9]. Having students follow a “recipe” is not ideal for teaching laboratory skills or scientific processes [10]. Furthermore, in traditional laboratories, since students are following a set protocol, they do not gain confidence in their ability to modify a protocol (“on‐the‐fly,” if necessary). As noted by Sinclair [4], “The ability to apply prior knowledge to new challenges is a skill that is highly valued by employers, but the confidence to achieve this does not come naturally to all students.” The ability to confidently design and adapt experimental methods are skills that are essential to a successful scientific career, but is not often explicitly taught in biology classes [11] and the literature suggests that many students graduate without these skills (see, for example, [12]).

Thus, there are at least two areas in which students could be further developed during their tertiary laboratory education.

1. Development of experimental laboratory *competence*. That is, the ability to design experiments, think critically and adapt protocols to suit specific needs.
2. Development of experimental laboratory *confidence*. That is, being confident in experimental method selection and protocol adaptation.

To improve development in these areas we here propose and then test a model for tertiary undergraduate laboratories based on the “Prepare, Do, Review” model [13], which is currently practiced in several units in our School (School of Molecular Sciences at UWA). Our goal is to improve a laboratory course based on the “Prepare, Do, Review” model by introducing a guided inquiry experiment (reviewed in [14]). Our new experiment introduces experimental design elements into each segment of the laboratory classes and in so doing helps students to think critically about laboratory methods, grow in their competence and confidence in experimental design and grow in their confidence to select, adapt (as necessary), and apply laboratory techniques to new challenges. One of the reasons “cookbook” style experiments are common in university education is that they are cost effective and easy to run. Hence, to improve the attractiveness of our approach, we aimed for our newly designed laboratory experience to be equally cost effective.

## 2 Methods/Experimental

### 2.1 Redesigning the Laboratory

The laboratory that we redesigned was one where students are required to separate a mixture of two proteins, ferritin (pI 4.8) and cytochrome C (pI 10.6), using cation exchange chromatography. They achieve this by diluting the supplied aqueous protein solution with a citrate buffer (pH 3.8), loading this dilution onto a column containing cation exchange resin and then passing buffers of increasing pH through the column to elute the bound proteins one at a time. The proteins in each fraction are then quantified by UV/Vis spectrophotometry. The full experimental protocol is available in the Supporting Information; this protocol is identical to the one followed by the students, except for minor formatting changes.

We modified each section of the “Prepare, Do, Review” model [13].

The “Prepare” segment involves consuming prelaboratory material (e.g., prerecorded PowerPoint presentations with background information, “how to” videos, extra readings, etc.) and completing an online quiz; in the “Do” segment, the students complete the laboratory exercise under competent supervision; the “Review” segment consists of a 1‐h group review session, during which several students give short presentations to the rest of the class, based upon questions posed in laboratory results sheets and the students then complete a postlaboratory quiz to cement their understanding of the laboratory. We redesigned all these segments to incorporate experimental design elements.

The “Prepare” segment was expanded to include three choices:

1. Which buffer should be used to dilute the protein mixture? Students were given six buffers to choose from: 0.01 M citrate (pH 3.8), 0.01 M citrate (pH 4.8), 0.05 M Tris (pH 5.8), 0.05 M Tris (pH 8.0), 0.05 M Tris (pH 9.0), 0.025 M phosphate (pH 12).
2. Which buffers should be used for elution? Students could choose any of the six buffers listed above.
3. In what order should the buffers be added?

Importantly, for choices 2 and 3, we did not specify how many buffers needed to be used. Students were at liberty to choose any buffer combination for completing the laboratory, but not all of the options will work equally well. To bind proteins with pIs of 4.8 and 10.6 to a cation exchange column, the loading buffer must have a pH of less than 3.8; to elute these proteins separately from the cation exchange column, the first elution buffer must have a pH between 5.8 and 10.6 and the second must have a pH greater than 11.6.

The “Do” segment was improved by allowing students to (a) follow the procedure that they designed in the “Prepare” segment and (b) adapt their protocol on‐the‐fly if they perceive that the experiment is not progressing as they had expected. Before beginning laboratory work, students were required to show written evidence that they had selected the buffers they wanted to use. Laboratory demonstrators were instructed to allow students to follow their protocol, even if the demonstrators knew that some students' methods would not achieve separation of the two proteins.

The “Review” segment was given a dual focus; the current focus on understanding and explaining results and observations was kept but we added to it opportunities for students to reflect on their experimental designs and discuss how well they worked with the rest of the group. Several students were selected to present answers to questions related to the laboratory to the rest of the group.

### 2.2 Assessing the Effectiveness of Our Redesigned Laboratory

Development of students' experimental design and protocol adaption competence was assessed by comparing their prelaboratory (“Prepare”) and postlaboratory (“Review”) scores for questions involving experimental design, through a postlaboratory survey (Tables 1 and 2) and analyzing their responses to a question that was answered during the laboratory session (“Do”). Prelaboratory quiz questions were all selected response; postlaboratory quiz questions used for our analyses were a mixture of matching and selected response questions. Survey responses were collected after completion of the laboratory in 2024 and students who completed the laboratory in 2023 or 2024 were invited to participate. The survey consisted of six statements about the laboratory (Table 1) and four constructed response questions (Table 2); students were instructed to indicate how much they agreed or disagreed with the statements on a scale of 1–5 and answer the four questions about the laboratory. The in‐laboratory question required a constructed response from students.

**TABLE 1:** Survey statements supplied to students after completing a second‐year undergraduate biochemistry laboratory session, which included experimental design elements. Students were asked to rate to what extent they agreed or disagreed with the statements on a 1–5 scale, with 1 being strongly disagree and 5 being strongly agree.

Statement number	Statement
1	The experiment provided me with an opportunity to take responsibility for my own learning
2	This laboratory helped develop my confidence to choose the right reagent for a particular laboratory procedure
3	This laboratory taught me how to adapt protocols for specific laboratory challenges
4	I enjoyed the freedom to choose which reagent I would use for my experiment
5	After this laboratory I am more confident in my ability to change a method to suit the needs of my experiment
6	I believe that the cognitive and practical skills I learnt in this laboratory session will be useful in my future career

**TABLE 2:** Survey questions supplied to students after completing a second‐year undergraduate biochemistry laboratory session, which included experimental design elements. Students were asked to give constructed responses to each of these questions.

Question number	Question
1	Please specifically provide comment on your experience in designing your own experiment as part of this laboratory
2	Did you enjoy doing the experiment? Why or why not?
3	What did you think was the main lesson to be learnt from the experiment?
4	What aspects of the experiment did you find the most enjoyable and interesting?

Data were analyzed using Microsoft Excel and the R statistical software (v4.3.0; R Core Team 2023) [15]. Survey responses were analyzed and visualized using the “tidyverse” R package [16]. R‐scripts are available at https://github.com/ndtivendale/biochemLabs. When analyzing students' constructed responses, responses that were clearly related to other elements of the unit (e.g., other laboratory exercises) were excluded.

### 2.3 Ethics

The survey and other assessments of laboratory redesign effectiveness were conducted under ethics approval 2024/ET000559 (UWA). Ethics approval was granted in accordance with the requirements of the National Statement on Ethical Conduct in Human Research (National Statement) and the policies and procedures of The University of Western Australia.

## 3 Results

### 3.1 Inclusion of Experimental Design Elements Increases Students' Experimental Laboratory Competence

**FIGURE 1:**
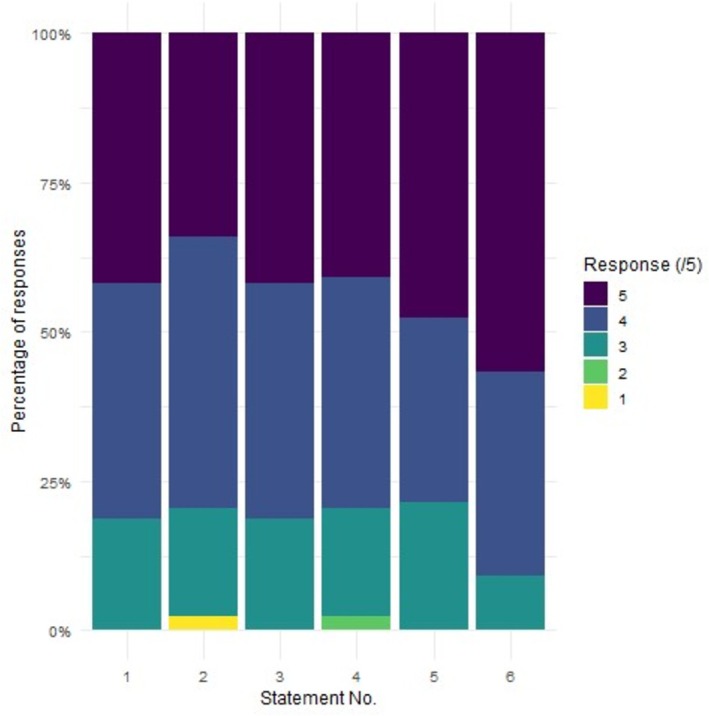
Responses indicating how much students agree or disagree with statements given in a survey that was offered to second‐year undergraduate students after completion of a biochemistry laboratory session. 1 = “strongly disagree,” 2 = “disagree,” 3 = “neutral,” 4 = “agree,” 5 = “strongly agree.” For statements 1–6, *n* = 43, 44, 43, 44, 42, and 44, respectively.

The development of students' laboratory confidence and competence was evident in all our analyses. We had a reasonable uptake rate for the survey, with 45 out of 184 eligible students (24%) completing the survey (not all students completed every question). The mean response to all six statements was “agree” (4) (Table 3). Median values were also four for all statements, except for statement 6, which addressed the usefulness of the skills learned in this laboratory exercise. The responses to these statements all had IQRs (interquartile ranges) of 4–5. More than 75% of respondents agreed or strongly agreed with all six statements (Figure 1). The survey was designed such that statements 3 and 5 addressed the students' self‐perceived laboratory competence. The scores indicated that the laboratory exercise increased the students' laboratory confidence and their self‐perceived competence.

**TABLE 3:** Responses to a survey given to students after completing a second‐year undergraduate biochemistry laboratory session, which included experimental design elements. 1 = “strongly disagree,” 2 = “disagree,” 3 = “neutral,” 4 = “agree,” 5 = “strongly agree.” Shown are means ± sd. For statements 1–6, *n* = 43, 44, 43, 44, 42, and 44, respectively.

Statement number	Statement	Response (/5)
1	The experiment provided me with an opportunity to take responsibility for my own learning	4.2 ± 0.8
2	This laboratory helped develop my confidence to choose the right reagent for a particular laboratory procedure	4.1 ± 0.9
3	This laboratory taught me how to adapt protocols for specific laboratory challenges	4.2 ± 0.8
4	I enjoyed the freedom to choose which reagent I would use for my experiment	4.2 ± 0.8
5	After this laboratory I am more confident in my ability to change a method to suit the needs of my experiment	4.3 ± 0.8
6	I believe that the cognitive and practical skills I learnt in this laboratory session will be useful in my future career	4.5 ± 0.7

In addition to students' self‐perceived competence, we measured competence by comparing their prelab and postlab scores for questions relating to experimental design. We included two types of experimental design questions in the pre‐ and postlab quizzes. The first type (type I) gives the student some information and then asks, in essence, “what should you do?” The second type (Type II) gives the student a scenario and then asks “what would happen?” One of each type of these questions was included in the pre‐ and postlab quizzes (Table 4) and comparing the success rate for these questions we can gage the students' increase in experimental laboratory competence. Comparing student performance in the pre‐ and postlaboratory quizzes showed a 21% increase for both the 2023 and 2024 cohorts' mean overall scores in the post‐laboratory quiz compared to the prelaboratory quiz. This indicates that the laboratory session may have positively influenced the students' understanding of the material as a whole (not just their experimental design capabilities).

**TABLE 4:** Prelab and postlab quiz questions used to assess students' experimental laboratory competence. Each question had three to four variants to minimize opportunities for cheating. Variation was introduced by changing the pI values of the proteins and the pH values of the buffers in the questions. *p*‐values were calculated using Z‐tests. When calculating the proportion of correct answers, those that achieved 75%–100% of available marks were considered correct; answers that scored less than 75% were considered incorrect.

Question type	Prelab quiz	Postlab quiz	Proportion correct (prelab)	Proportion correct (postlab)	*p*
I. What should you do?	You wish to isolate a protein of pI *x* by ion exchange chromatography as quickly and efficiently as possible, from a mixture that also contains proteins of pI *y* and pI *z* (in some variants: protein of pI *y*). Select the most correct method.	You have a protein mixture containing two proteins and a small molecular weight contaminant. Protein A has a pI of *x* and protein B has a pI of *y*. You want to separate the two proteins and remove the contaminant. You know that the contaminant has a pI of *z*. Using an anion exchange column, what pH buffers would you choose for the following actions. Answer the statements below by selecting the single most appropriate option from the answer options list below. Any of the options may be correct once, more than once, or not at all.	0.55 (2023) 0.47 (2024)	0.80 (2023) 0.88 (2024)	1.3 × 10^−3^ (2023) 1.4 × 10^−8^ (2024)
II. What would happen?	Using an anion instead of a cation exchange resin (in some variants: a cation exchange resin) and all other materials as described in the lab notebook, if you prepare your column and dilute your protein sample in *x* buffer (pH *y*), which of the following would happen?	Imagine you are given a mixture containing cytochrome C and ferritin and an unknown protein with a pI of *x*. You want to purify ferritin (in some variants: Cytochrome C) from the mixture using a cation (in some variants: anion) exchange column and one or more of the buffers supplied in this laboratory session. What would happen if you prepared the column and loaded the sample in Tris buffer (pH *y*)?	0.62 (2023) 0.85 (2024)	0.70 (2023) 0.80 (2024)	0.38 0.39

Improvements in the students' laboratory competence as it relates to experimental design were measured by comparing the changes in the proportion of correct responses in the pre‐and postlab quizzes. Interestingly, this analysis showed a significant improvement in students' competence in answering Type I questions but no statistically significant change in their competence in answering Type II questions. This suggests that our guided inquiry laboratory improved the students' ability to conceptualize good experimental protocols (what should you do?) but had no significant effect on their ability to critically assess and predict the outcome of a particular experimental protocol (what would happen?).

In 2023, students' experimental design competence could also be seen in their responses to a constructed response experimental design question that was answered by each student during the laboratory session. The question was: “Imagine you are given a new mixture of two proteins, Protein A with a pI of 6.8 and Protein B with a pI of 9.8. You know the mixture also contains a contaminating Protein C with a pI of 11. Fill in the table below to show how you would use an anion exchange column to separate these proteins and remove the contaminant.” The mean score for this question was 1.49/2 (± 0.4; sd), with 33% scoring full marks and 99% scoring a pass mark (1/2) or above. Students generally displayed in‐depth understanding of ion exchange chromatography in this question and demonstrated their ability to take theoretical concepts (e.g., the charge of a protein with a certain pI at a certain pH) and apply them to design laboratory procedures. This question also probed the students' understanding of two different kinds of ion exchange resin (anion vs. cation).

### 3.2 Inclusion of Experimental Design Elements Increases Students' Experimental Laboratory Confidence

Our aim in this laboratory was to develop students that are both competent and confident in their experimental design capabilities. There is a clear link between competence and confidence [17] so, in light of the above results, it was unsurprising that students' confidence also increased as a result of this laboratory. Statements 1, 2, 4, and 6 in the survey gave insight into students' feelings of laboratory confidence or lack thereof and on average, students agreed with these survey statements (Table 3, Figure 1).

The increase in students' confidence in experimental method selection was most clearly seen in the students' answers to the constructed response questions (Table 5). Between 56% and 64% of survey respondents responded to these questions. A common theme in the responses was that the students initially lacked confidence in their ability to choose the “right” buffers for the experiment. One student described the experience of designing their own experiment as “…a little bit scary, since…we were just left to do it on our own whether it was right or wrong”; another said the laboratory session was “a learning curve”; and a third “…was worried about whether [they were] doing it right.” However, many students said that by the end of the laboratory, they were glad they had been forced to decide which buffers to use and in what order. One student commented that the laboratory “…was challenging [sic], but also satisfying to be able to have a go in designing the experiment.” Two students highlighted the doubts that many scientists feel when they begin designing their own experiments, writing “…designing the experiment was stressful…” and that they were “doubting” themselves during the laboratory exercise. These comments indicate that some students exposed to this kind of laboratory experience grow in laboratory confidence because of it.

**TABLE 5:** Selected student comments from a survey supplied to students after completing a second‐year undergraduate biochemistry laboratory session, which included experimental design elements.

Comment
It was a little bit scary, since previously we have been given feedback on our choices (i.e., in BIOC2001) but this time we were just left to do it on our own whether it was right or wrong. I think I would have liked the reassurance on my choices before starting. But that's probably only because I don't like wasting resources
Fun exciting. Confusing at times but good learning experience overall
It provided a direct link between how we understood the background information, and it's application to a lab setting
It let me see the direct effect that changing parts of protocol had on experimental outcomes; for example I saw how having a ph too close to the pI didn't result in elution of a desired band, and I ended up seeing that band elute just as another was eluting when adding my final buffer, though this wasn't ideal it greatly contributed to my understanding.
Overall, designing my own experiment was a good experience, but it was sometimes challenging. Understanding certain topics proved difficult at times, which made it hard to create a well‐structured experiment. Despite these challenges, I learned a lot about problem‐solving and how to approach complex topics more effectively
It challenges me to apply the skills from prelab lectures into the theory required for practicals
Enjoyed it very much, led to greater understanding of protein charge states
The level of independence was good as it required me to specifically think how each reagent will affect the experiment
I found this a lot harder than past experiments as there was a lot more understanding needed that although in the laboratory materials did not make much sense to me. I was able to complete the tables needed before the lab but was doubting myself as it seemed almost too simple given the topic of the lab
It was great to design our experiment as it helped to understand the possible errors and possible methods
Yes, I enjoyed doing the experiment because it allowed me to apply what I've learned in a practical way. It was satisfying to see the results of my work and to overcome the challenges I faced along the way
It was enjoyable, because the element of autonomy was appreciated, however because it was a “once ‐off,” and all other labs had been guided, it was odd that this one was the only one where it was slightly less guided. It wasn't enjoyable for the fact that it wasn't clear how to set up this experiment.
I did not enjoy this experiment at first, it felt like a huge jump from the other lab sessions. But looking back it was very beneficial to my course progression
Yes—felt more in control and less rushed/confused than in other labs and I found it to provide me with greater understanding of laboratory techniques
Thoroughly enjoyed this experiment, as there was a bit more autonomy provided. There was a drawback to this as well. One of the decisions I made, produced an imperfect result. My pair‐partner was distressed. I would've preferred full autonomy to make mistakes, and would've accepted full responsibility too that is, individual work and a lower mark. However, as a starter step, this laboratory gave me the confidence to go ahead and choose a L3 BIOC unit in 2024
I found the most enjoyment in designing the experiment and seeing the results come together. It was interesting to apply theoretical concepts to a real‐world scenario and watch how different variables interacted. I also appreciated the problem‐solving aspects, which allowed me to think critically and adjust the experiment as needed

*Note:* Where the intended meaning was clear, grammatical and spelling errors in student comments have been corrected.

Survey respondents also reported enjoying the experimental design elements of the laboratory. One student enjoyed “…having the freedom to choose…”; another said that “…designing the experiment was…more interesting [than just following instructions]”; and a third “…enjoyed…having a moderate level of independence.” The laboratory was variously described by students as “fun,” “exciting,” “engaging,” and “excellent.” The students' enjoyment of the laboratory was also reflected in the survey responses to question 4 (Table 3), where most students agreed that they “…enjoyed the freedom to choose which reagent [they] would use for [their] experiment.”

Nevertheless, the comments also showed that some students did not understand that we were giving them more autonomy to teach them inquiry‐based skills. One respondent would “have liked the reassurance on [their] choices before starting.” and another commented that “…it wasn't clear how to set up this experiment.” This highlights the fact that some students were unprepared for taking on inquiry‐based learning in their undergraduate classes and expected this laboratory to be more like the other laboratories they had experienced. It may be that embedding inquiry‐based learning into curricula more widely (e.g., into lecture and tutorial classes) would help students become more comfortable with this approach. These comments may also demonstrate that these particular students had not taken the time necessary to engage with the prelaboratory material and so were ill‐prepared for making the choices required by this laboratory.

The ability to design experiments requires an understanding of the relevant theory. One respondent emphasized this, writing “The level of independence was good as it required me to specifically think how each reagent will affect the experiment”; another commented that the experimental design elements of the laboratory meant that they “Had to think…[, which] made [their] learning better.” And a third wrote “I found…there was a lot more understanding needed….” It is probable that the inclusion of experimental design elements in this laboratory provided an impetus for this student and others to engage deeply with the rest of the prelaboratory material and thereby gain a better understanding of the theory. This was an extra benefit of our improved laboratory session and highlights again the link between confidence and competence.

## 4 Discussion

In this study we tested a method of improving students' experimental laboratory competence and confidence by incorporating experimental design elements into a second‐year undergraduate biochemistry laboratory session. We found that students generally increased in both experimental laboratory confidence and competence after completing the laboratory. Our findings are consistent with the results reported by Carmel et al. [18], who found that inquiry‐based laboratory experiences, in contrast to protocol‐based experiences, offer greater opportunity to develop and use scientific research skills (as defined by the National Academies; [19]).

For traditional, protocol‐based laboratory classes, students follow a set protocol with success in the laboratory depending upon a student's ability to follow written instruction and technical laboratory competence. Although valuable for teaching students basic laboratory skills, this structure does not enable students to develop: (1) skills in thinking critically and adapting protocols to suit specific needs (experimental laboratory competence) and (2) confidence in experimental method selection and protocol adaptation (experimental laboratory confidence). We successfully introduced these skills by modifying an existing laboratory. This is an advantage when there is limited funding and time available to design and deliver new laboratories.

The short duration, and associated cost savings, are an important point of difference between the inquiry‐based laboratory experience reported here and typical course‐based undergraduate research experience (CURE), which usually takes 9–16 weeks [20]. Nevertheless, the results that we observed were similar to those observed when CUREs are implemented. For example, Chaari et al. [21] reported that implementation of a biochemistry CURE allowed students to improve and develop content knowledge and Jones et al. [22] saw an increase in students' confidence to design experiments and construct hypotheses after completing a biochemistry CURE. It should be noted that the type of confidence built by inquiry‐based laboratories like the one we report here and CUREs is different from gaining confidence in performing basic techniques, which is better served by no‐stakes laboratory training workshops [23]. Another advantage of a CURE that carried over into our inquiry‐based laboratory is that students develop technical communication skills [21].

One somewhat surprising finding from our study was that students enjoyed and appreciated inquiry‐based learning. Others have also reported on this. For example, Roller et al. [14], reported the development of a set of seven undergraduate guided‐inquiry practical chemistry exercises to allow students to learn laboratory skills. The so‐called MICRO (Making Introductory Courses Real while Online) laboratories were introduced remotely and in‐person in 2020 at nine institutions. Just as students in our study enjoyed and appreciated the inquiry‐based nature of the laboratory, feedback from students in the Roller et al. [14] study indicated the same. They also liked that the laboratory forced them to ask questions, solve problems, and engage in experimental design, something the students had not been so engaged with in previous semesters. In a similar way, in our study, one student commented that they appreciated the opportunity to participate in experiential design, as this forced them to gain a better understanding of the theory behind the laboratory.

Other groups have reported on the effectiveness of inquiry‐based approaches for not only improving students' experimental design capabilities and confidence but also their understanding of the course material. For example, Bugarcic et al. [24] showed that an inquiry‐based undergraduate laboratory on cell culture improved students' basic understanding of molecular and cellular processes, as well as their understanding of experimental design. Similarly, Nag [25] showed that inquiry‐based learning biochemistry laboratory experiences correlated with higher assessment scores compared to cookbook‐style laboratories. Similarly, our results suggest an improvement to the students' understanding of the course material (Table 4).

An important aspect of our inquiry‐based laboratory model is that students are required to engage in experimental design and then execute the experiment they designed, with little input from laboratory demonstrators. In the laboratory exercise reported here, students were required to select appropriate buffers for separating two proteins, but not all the buffer combinations would lead to a successful separation. Even if students' designs were not going to be successful in separating the two proteins, laboratory demonstrators were instructed not to offer any guidance in this area. Students were allowed to carry out their experimental protocols, even if they were not going to be successful. Allowing students to do this is a form of “productive failure,” which is argued to be important in science, technology, engineering, and mathematics education [26, 27]. It was made clear to the students that their marks for the assessments associated with the lab did not depend upon a successful separation, which provided a “low stakes” learning environment.

One of the challenges with teaching undergraduates using inquiry‐based laboratory exercises is increased cognitive load, which is the mental effort required to learn new material [28]. In some respects, our transformed laboratory increases the cognitive load for students because they not only have to learn fundamental concepts in the “Prepare” segment of the laboratory, but they also must apply that new knowledge to make experimental design decisions. We manage this increased cognitive load in the “Prepare” segment by introducing the fundamental concepts through a video recorded lecture and written materials before asking students to decide which buffers to use. Nevertheless, if students have not adequately understood the fundamental prelaboratory material, then they will likely select inappropriate buffers for the experiment and therefore be confused or frustrated when their choices result in an ineffective separation. For these students, perhaps the cognitive load was too high. Hence, we also manage the increased cognitive load in the “review” segment, where there is time and space to reflect and discuss experimental designs, which ones worked or did not and why they did or did not work. Although inquiry‐based laboratories increase cognitive load, they also encourage deep engagement with theoretical material (as outlined in the Results section above). Furthermore, inquiry‐based laboratories like ours are a form of active learning, which has been shown to improve understanding of fundamental biochemical concepts [29].

Another common challenge with inquiry‐based undergraduate laboratories is scalability, because it becomes harder for teachers to support individual students in larger classes. However, our laboratory is readily scalable because it does not require substantial amounts of input on an individual student basis. Students are supported in the “prepare” segment through the online course materials and lecturers are readily contactable to address specific areas of confusion. In the “do” segment, students are split into multiple groups and supported by laboratory demonstrators, who guide them through the technical aspects of the laboratory, although they will not comment on their experimental design. In the “review” segment, students are, once again, split into multiple groups and supported by small and large group discussions (with each large group attending one session), during which any residual confusion about the laboratory exercise can be clarified. For each of these steps, we do not foresee any difficulty in scaling up this laboratory exercise, beyond the difficulties normally associated with scaling up, such as the increased faculty teaching load and increased number of laboratory demonstrators required.

One of the keys to developing students into competent and confident scientists is to improve their ability to apply prior knowledge and principles to new situations. Sinclair [4] reports the development of a series of three laboratory classes for bioscience teaching that go beyond the following of fail‐safe recipes and require in‐the‐moment decision‐making and the recall of skills and their adaption to new challenges in the laboratory. Sinclair notes that “The ability to apply prior knowledge to new challenges is a skill that is highly valued by employers, but the confidence to achieve this does not come naturally to all students.” The author assessed success of the newly developed laboratories by asking students to report their level of confidence in eight key employability traits before and after completing the classes. After completing the laboratories, the proportion of students reporting a high level of confidence in these traits rose by between 9% and 35%. The traits assessed included problem solving, self‐motivation and “ability to learn and adapt.” These three traits in particular are part of what we would call scientists' experimental laboratory competence and if competence is achieved in these areas it would lead to the type of confidence increase reported by Sinclair [4]. Nevertheless, self‐reported increases in confidence do not necessarily imply cogent increases in competence. Therefore, while this study shows that the types of laboratory experiences reported by Sinclair help students to gain confidence in the laboratory, it does not necessarily show that students' competence in the laboratory increased also. We have extended the findings of Sinclair [4] by demonstrating that students' experimental competence as well as experimental confidence increases when students are required to exercise their own judgment in the laboratory.

## 5 Conclusions

Laboratory classes offer an excellent environment for active learning and developing skills preferred by employers. In this study, we introduced active learning into a second‐year undergraduate protein purification laboratory by converting it from a cook‐book style laboratory exercise into a guided inquiry. We showed that our guided inquiry laboratory improved students' ability to determine what should be done given a specific laboratory scenario. Our transformed laboratory also led to a gain in students' confidence to select and adapt protocols to suit a particular situation. Furthermore, students on the whole reported enjoying engaging with experimental design.

## Author Contributions

N.D.T. conceived of the improvements to the laboratory and all authors developed and implemented the improved laboratory. All authors designed the measures of success and A.V.D. and P.G.A. implemented them. N.D.T. analyzed the data. N.D.T. and P.G.A. wrote the manuscript. All authors approved the manuscript for publication.

## Funding

The authors have nothing to report.

## Conflicts of Interest

The authors declare no conflicts of interest.

## Supporting information


**File S1:** Experimental protocol.

## Data Availability

All data generated or analysed during this study, except the raw data, are included in this published article. The raw data cannot be made publicly available due to privacy or ethical restrictions.
