# Supplementary material for: Introducing Experimental Design Concepts Improves Biochemistry Undergraduates' Experimental Laboratory Competence and Confidence
Source: Biochem Mol Biol Educ. 2026 Mar 14;54(3):254–63. doi: 10.1002/bmb.70046 (PMC13206386; doi:10.1002/bmb.70046)
Supplement: Supplementary file 1 — File S1: Experimental protocol. [file BMB-54-254-s001.docx]

Equipment and Reagents

1. Resin - Carboxymethyl cellulose. Gel slurry suspended in citrate buffer (pH 3.8). Resuspend by mixing immediately before use.
2. 0.05 M Tris buffer (pH 5.8)
3. 0.025 M phosphate (pH 12)
4. 0.01 M citrate buffer (pH 3.8)
5. 0.05 M Tris buffer (pH 8.0)
6. 0.01 M citrate buffer (pH 4.8)
7. 0.05 M Tris buffer (pH 9.0)
8. Concentrated protein mixture (3 mg/ml) - available from demonstrator.
9. Boiling tubes (50 ml) - drawer in laboratory
10. Test tubes (20 ml) - drawer in laboratory
11. 10 mL measuring cylinder
12. Plastic chromatography column
13. Pasteur pipettes
14. Gilson P1000 pipettes
15. Pipette tips (blue)
16. BMG FLUOstar OMEGA plate reader
17. Microtitre plates

Important! Experiment Design

**Prior to commencing the lab**, as part of your pre-laboratory work you will need to plan the experiment (as a researcher would). Choose the buffers you will use to separate ferritin (pI 4.8) and cytochrome C (pI 10.6) from a mixture of the two proteins, using the carboxymethyl cellulose resin as the stationary phase of this ion-exchange column. You may not need to use every buffer supplied and you may not need to use 5 elution buffers. 

***Your demonstrator will be checking that you have designed an experimental procedure in the following table before you begin your lab work***

| **Table 1** | |  |
| --- | --- | --- |
|  | Buffer | Protein you expect to elute |
| Dilution of protein mixture |  |  |
| First elution |  |  |
| Second elution |  |  |
| Third elution |  |  |
| Fourth elution |  |  |
| Fifth elution |  |  |

Experimental Procedure

Working individually in this experiment you will be required to set up an ion-exchange column and use this column to separate ferritin (pI 4.8) and cytochrome C (pI 10.6) from a mixture of the two proteins. You will then determine the percentage of the mixture that each protein comprised and the overall recovery for the experiment.

**Read all parts before proceeding**

| ***Health and Safety***   - Normal laboratory PPE (i.e. laboratory coats, closed toe shoes, long hair tied up) should be used. - Review the reagents and experimental procedure to determine if gloves are required in this experiment. |
| --- |

***Protocol - Preparation of ion exchange column***

******WARNING!! Do not let your gel dry out******A dry gel will affect protein separation. Always have liquid above the top frit.

1. Prepare the plastic column for use by removing the bottom cap and making sure the bottom frit is flat.  **#** See column setup in "Extend your learning resources".

2. Add water until the frit is wet and water starts dripping from the column.  **#** Give the column a sharp tap if it does not flow. See demonstrator if it still does not flow.

3. Allow 3-4 mL to flow through to wash the frit. **#** Cap the column to stop the flow. It is a good idea to keep a collection tube under the column at all times to avoid creating a mess.

4. Remove the excess water with a Pasteur pipette leaving a layer of about 1-2 mm above the frit.

5. Check that there are no bubbles below the frit as they can reduce the flow rate dramatically.

6. Gently resuspend the resin. **#** See column setup in "Extend your learning resources".

7. Pour all of the resin suspension into the column to give a settled gel height of about 1.5 cm.

8. Allow the column to run until the liquid layer is just filling the narrow part of the column. Cap the bottom of the column.

9. Gently apply a frit to the top of the gel and then let the liquid layer run down to within 1-2 mm of the surface of the frit.

10. Add 2 mL of the appropriate buffer (the buffer you have made *protein 2* up in) very carefully to the column making sure you do not stir up the gel. **#** If you do stir up the gel, let the gel resettle before continuing.

11. Remove the bottom cap and allow the buffer to run through the column until the top of the liquid layer is about 2 mm above the surface of the gel and then replace the bottom cap.  **#** You are ready to separate the protein mixture.

***Protocol - preparation of the protein sample***

1. Aliquot 700 μL of the protein mixture into a clean test tube and dilute it in water to a final volume of 7 mL. Label it *"Protein 1”* and set aside.
2. Aliquot another 700 μL from the remainder of your protein mixture and dilute it with your chosen buffer to a final volume of 7 mL, label it *“Protein 2”* and set aside.

***Running the Column***

***DO NOT THROW AWAY ANY ELUTED SAMPLES.**

1. Collect a) an appropriately sized test tube rack, and b) glass test tubes for each buffer you are using in your experimental procedure.  **#**Consider the volumes you require.
2. Label with a marker each glass test tube appropriately before collecting your buffers.
3. Carefully pipette 5 mL of *Protein 2* (not *protein 1*) into your column.
   1. Remove the bottom cap and allow the solution to run through into the 10 mL measuring cylinder until the top of the liquid layer is just above the surface of the frit (2-3 mm). This is the first fraction (*Fraction 1*).
   2. Replace the bottom cap and record your observations in Table 3 **#** In-lab Experimental Record Question 1.
   3. Make the eluate (i.e. the liquid that flowed out of the column) collected in the measuring cylinder up to 6 mL using the appropriate buffer. Then transfer the diluted eluate into a clean, labelled test tube and put aside. Rinse your measuring cylinder with deionised water.
4. Add 1 mL of the first elution buffer to the column.
   1. Remove the bottom cap and collect the effluent in the washed 10 mL measuring cylinder.
   2. When the top of the liquid layer is just above the surface of the frit add a further 3 mL of the same buffer.
   3. Allow this to run through your cylinder until the top of the liquid layer is just above the frit (*Fraction 2*) and then replace the cap on the bottom of the column. Record your observations in Table 3.
   4. Make the eluate up to 6 mL with the appropriate buffer, transfer to a labelled tube and put aside. **#**Use stock buffer solution to make up to 6 ml.
5. Record your observations in Table 3.
6. If necessary, repeat step 4 with the second (*Fraction 3*), third elution buffers (*Fraction 4*), and so on. Record your observations in **Table 3**.  **#** You may not need to use each of the buffers supplied for your method, but others in the class may have chosen to use each of the buffers, so you may end up with a different number of fractions to some of your peers.

**Measuring protein concentration.**

1. Pipette 200 μL of water, in triplicate, into the plate to measure the background. **#** This will be the background absorbance without protein.
2. In triplicate, carefully pipette 200 μL of Protein 1 solution into a 96-well plate (template below).
3. In triplicate, carefully pipette 200 μL of each of your collected eluates (*Protein 2* and *Fractions*) into a 96-well plate.
4. Record the absorbance measures in **Table 4.**

|  |
| --- |

Suggested template below. Note, you may have fewer fractions depending on how you set up the experiment. If you have more fractions continue the template in columns (e.g. 9, 10, etc.).

| ***Table 2*** | | | | | | | | | | | | |
| --- | --- | --- | --- | --- | --- | --- | --- | --- | --- | --- | --- | --- |
|  | **1** | **2** | **3** | **4** | **5** | **6** | **7** | **8** | **9** | **10** | **11** | **12** |
| **A** | Water | Protein 1 | Fraction 1 | Fraction 2 | Fraction 3 | Fraction 4 | Fraction 5 | Fraction 6 |  |  |  |  |
| **B** | Water | Protein 1 | Fraction 1 | Fraction 2 | Fraction 3 | Fraction 4 | Fraction 5 | Fraction 6 |  |  |  |  |
| **C** | Water | Protein 1 | Fraction 1 | Fraction 2 | Fraction 3 | Fraction 4 | Fraction 5 | Fraction 6 |  |  |  |  |
| **D** |  |  |  |  |  |  |  |  |  |  |  |  |
| **E** |  |  |  |  |  |  |  |  |  |  |  |  |
| **F** |  |  |  |  |  |  |  |  |  |  |  |  |
| **G** |  |  |  |  |  |  |  |  |  |  |  |  |
| **H** |  |  |  |  |  |  |  |  |  |  |  |  |

***Good Lab Citizens****- Clean up:*

- Buffers and used samples in tubes and microtitre plate can be flushed with water down the laboratory sinks.
- Remove marker from glass wear using ethanol.
- Place column (assembled), Pasteur pipettes, rinsed glassware and microtitre plate in the appropriate labelled containers or space on the laboratory sink.
- Return racks to cupboards.
